# Fifteen Years After Sleeve Gastrectomy: Weight Loss, Remission of Associated Medical Problems, Quality of Life, and Conversions to Roux-en-Y Gastric Bypass—Long-Term Follow-Up in a Multicenter Study

**DOI:** 10.1007/s11695-021-05475-x

**Published:** 2021-05-22

**Authors:** Daniel M. Felsenreich, Evi Artemiou, Katharina Steinlechner, Natalie Vock, Julia Jedamzik, Jakob Eichelter, Lisa Gensthaler, Christoph Bichler, Christoph Sperker, Philipp Beckerhinn, Ivan Kristo, Felix B. Langer, Gerhard Prager

**Affiliations:** 1 Division of Visceral Surgery, Department of General Surgery, Medical University of Vienna, Waehringer Guertel 18-20, 1090 Vienna, Austria; 2 Department of Surgery, Hospital Rudolfstiftung, Vienna, Austria; 3 Department of Surgery, Hospital Hollabrunn, Hollabrunn, Austria

**Keywords:** Sleeve gastrectomy, Weight regain, Conversion, Quality of life, Roux-en-Y gastric bypass, Associated medical problems

## Abstract

**Purpose:**

Since 2014, sleeve gastrectomy (SG) has been the most frequently performed bariatric-metabolic operation worldwide (2018: 386,096). There are only a few studies reporting a long-term follow-up (up to 11 years) available today. The aim of this study was to evaluate the long-term outcome of SG with a follow-up of at least 15 years regarding weight loss, remission of associated medical problems (AMP), conversions, and quality of life (QOL).

**Setting:**

Multicenter cross-sectional study; university hospital.

**Methods:**

This study includes all patients who had SG before 2005 at the participating bariatric centers. History of weight, AMP, conversions, and QOL were evaluated by interview at our bariatric center.

**Results:**

Fifty-three patients met the inclusion criteria of a minimal follow-up of 15 years. Weight and body mass index at the time of the SG were 136.8kg and 48.7kg/m^2^. Twenty-six patients (49.1%) were converted to Roux-en-Y gastric bypass (RYGB) for weight regain and gastroesophageal reflux within the follow-up period. Total weight loss after 15 years was 31.5% in the non-converted group and 32.9% in the converted group. Remission rates of AMP and QOL were stable over the follow-up period.

**Conclusion:**

Fifteen years after SG, a stable postoperative weight was observed at the cost of a high conversion rate. Patients converted to RYGB were able to achieve further weight loss and preserve good remission rates of AMP. SG in patients without the need of a conversion to another bariatric-metabolic procedure may be considered effective. Careful preoperative patient selection is mandatory when performing SG.

**Graphical abstract:**

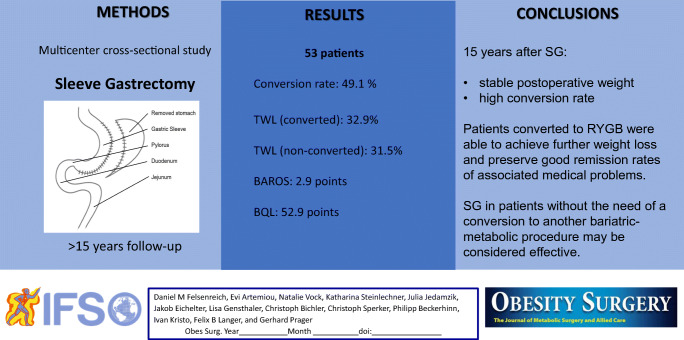

## Background

Obesity is a disease that continually increases in numbers worldwide. It is strongly associated with numerous medical problems of the metabolic syndrome [1]. Today, several bariatric/metabolic operations are available, and the number of performed procedures globally increases each year [2]. Since 2014, sleeve gastrectomy (SG) has been the most frequently executed operation for patients with obesity worldwide. In 2018, 386,096 (55.4%) patients underwent SG [3].

In its beginning, SG, a mainly restrictive operation which entails the resection of a greater part of the stomach, was a component of biliopancreatic diversion with duodenal switch [4]. Later, SG was mostly performed in patients with severe obesity (BMI >50kg/m^2^) as the first step of a two-step procedure. A second bariatric-metabolic procedure would be performed after initial weight loss [5]. Currently, SG is an accepted stand-alone bariatric-metabolic procedure for most patients with obesity [6].

Studies reporting a short-term follow-up after SG usually present good weight loss, remission of associated medical problems (AMP), and only low numbers of patients with weight regain (WR) and gastroesophageal reflux disease (GERD) [7]. In studies with a longer follow-up period, an increasing number of patients suffer from WR and GERD, some of them in need of a conversion to another bariatric-metabolic operation [8–10].

The efficacy of a bariatric-metabolic procedure may only truly be evaluated at a long-term follow-up. However, there are just a few studies reporting a long-term follow-up (up to 11 years) available in the literature today [11, 12]. Thus, the aim of this study was to evaluate patients that received SG before 2005 with a follow-up of at least 15 years in a multicenter setting. Assessed variables are the history of weight, remission of AMP, conversion to other bariatric-metabolic procedures, and the patients’ quality of life (QOL).

## Patients and Methods

All patients that underwent SG for obesity before December 2005 in one of three Austrian bariatric centers were included in this multicenter study. The participating centers were Medical University of Vienna, Hospital Rudolfstiftung in Vienna, and Hospital Klosterneuburg.

This study was approved by the Review Board of the ethical committee of Medical University of Vienna (Reference number: 2169/2019). Informed consent was obtained from all participating patients.

### Patient Cohort

The included collective represents a 15-year follow-up of the same 53 patients reported by Felsenreich et al. in 2016 (10-year follow-up after SG) [13]. Each patient had a preoperative gastroscopy ensuring that none of the included patients had Barrett’s esophagus or hiatal hernia. Participants did not have symptomatic reflux either at the time of the SG.

Patients who still have their SG after 15 years (non-converted patients), as well as patients who were converted within the follow-up period, were interviewed on their history of weight, remission of AMP, and conversions to other bariatric-metabolic procedures. Additionally, non-converted patients were asked to complete the following questionnaires on the outcome after bariatric-metabolic surgery and QOL: Bariatric Analysis and Reporting Outcome System (BAROS) [14], Bariatric Quality of Life Index (BQL) [15, 16], and Short Form 36 (SF-36) [17].

To achieve a high follow-up rate, patients were contacted per telephone or mail and called in for an interview. In Austria, the current home address can be acquired from the registration offices for scientific reasons as well as data from the obituary column to avoid patients lost to follow-up. Data of deceased and acutely converted patients (within a short period of time after the SG) were included in the baseline characteristics but not in the further calculation of the follow-up.

### Surgical Technique

**Fig. 1:**
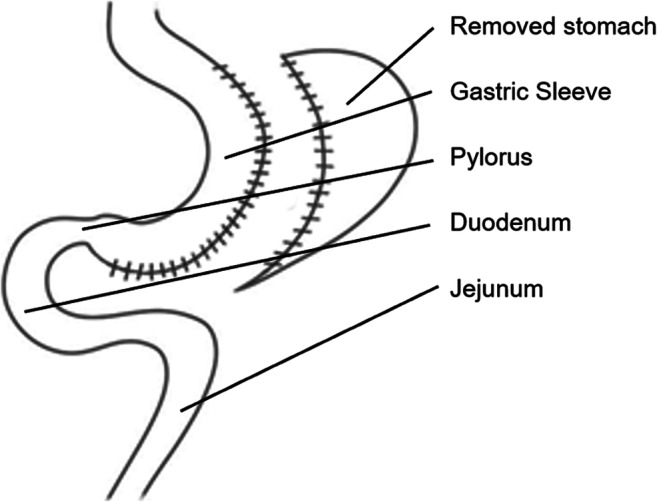
Sleeve gastrectomy

The technique of performing a SG (Fig. 1) was similar in the three bariatric centers participating in this study and will briefly be described in this chapter. After creating a capnoperitoneum with 12–15mmHg, the lesser sac was entered and opened from the pylorus to the angle of His at the greater curvature of the stomach while preserving the gastro-epiploic arcade. The sleeve’s blood supply is ensured by the right and the left gastric arteries. The left crus of the hiatus was visualized to detect undiagnosed hiatal hernias. Any hiatal hernias detected during this step would have been treated with hiatoplasty at this point. However, in this series, none of the patients had any hiatal hernia detected intraoperatively. Then, a 14–16mm (42–48 French) bougie was inserted to guide and standardize the resection of the stomach using five to seven stapler magazines. The starting point was at about 6cm distance from the pylorus and stapling ended at the angle of His to resect the entire fundus. Belsey’s fat pad was removed to increase the vision to the angle of His. The staple line was oversewn by a running suture.

The technique of performing a SG common then has only changed slightly up until now. The differences are that today, we use a slightly smaller bougie size of 12mm (36 French) and start the resection slightly closer to the antrum (2–3cm from the pylorus). The complete resection of the entire fundus was performed then and is still done today [18].

### Weight Loss

Weight and BMI (body mass Index) at the time of the SG (as well as at the time of the conversion in converted patients) were gathered from operation protocols. Patients were asked for their nadir weight (including point in time when it was reached) after SG (and after conversion, if applicable). Data on patients’ 10-year postoperative weight was published in the above-mentioned publication for the same cohort [13]. As before, weight regain was defined as weight gain of >10kg from nadir weight [13]. Patients’ current weight 15 years after SG was determined by weighing them in our walk-in clinic for standardized values from verified scales.

### Conversion to Other Bariatric-Metabolic Procedures

All operation protocols and electronic patients’ charts were studied to gather information about the reason for the conversion, body weight, status of AMP, etc.

### Remission of Associated Medical Problems

The status of AMP at the time of the SG (as well as at the time of the conversion in converted patients) was gathered from the operation protocols. The status of AMP at 10 years postoperative was published before [13], and for the current status 15 years after SG, patients were interviewed at our outpatient clinic. Remission or new onset of AMP was defined by an (discontinued/new) intake of disease-specific medication.

### Quality of Life

The outcome of the SG and the QOL at the long-term follow-up were both evaluated using questionnaires (BAROS, BQL, and SF-36) only in non-converted patients.

BAROS is a validated tool to evaluate the outcome of bariatric-metabolic surgery. It consists of five categories (weight loss, improvement of AMP, QOL, complications, and reoperation) the outcome of which is classified as failure, fair, good, very good, or excellent [14].

The BQL is a user-friendly questionnaire to gather information about patients’ QOL after bariatric-metabolic surgery. It consists of 13 questions, with a maximum total score of 65 points. It creates a comprehensive picture of patients’ QOL in their daily routine [15, 16].

SF-36 is a validated general QOL score including 36 questions that represent patients’ QOL in 8 different categories that focus on physical (physical functioning (PF), physical role (RP), bodily pain (BP), general health (GH)) and mental QOL (emotional role (RE), mental health (MH), vitality (VT), social functioning (SF)) [17].

### Statistical Analysis

The presentation of data in this study was as percentage, by mean and standard deviation (SD), or median and range (R). Comparing different groups of data, either non-parametric Mann-Whitney U test or chi-square test was used. Univariate analysis was two-tailed with statistical significance defined as a p-value <0.05. Excess weight loss (EWL) in % was calculated based on a BMI of 25kg/m^2^ (upper limit of the ideal BMI). The number of participants included in this study was limited by the number of patients that were able to reach the follow-up of at least 15 years. Data was collected in Excel for Windows (Microsoft, Redmond, Washington, USA), and statistical calculations were performed using SPSS V24 for Windows (IBM Corporation, Armonk, NY, USA).

## Results

Fifty-three patients received SG as a bariatric-metabolic procedure before December 2005 in any of the three participating centers, and, therefore, the follow-up of this study is at least 180 months. The patients’ characteristics are highlighted in Table 1. Previous operations were adjustable gastric banding in ten patients (18.9%), gastric stimulation in one patient (1.9%), and an endoscopic gastric balloon placement in one patient (1.9%). Forty-one patients (77.3%) had SG as primary bariatric-metabolic procedure.

**Table 1:** Patient characteristics

	*All patients (SG)*
	(n=53)
Sex (female) (n=42)	79.0%
Bariatric-metabolic procedures before SG (n=12)	22.6%
Gastric banding (n=10)	18.8%
Gastric stimulation (n=1)	1.9%
Gastric balloon (n=1)	1.9%
Converted patients (n=26)	49.1%
RYGB (n=25)	47.2%
Duodenal switch (n=1)	1.9%
Reasons for conversion	
GERD (n=10)	18.9%
Weight regain (n=14)	26.4%
Acute conversion (n=2)	3.8%
Median interval SG – conversion (n=24*) (in months)	48 (R 12-175)
Dead patients (n=4)**	7.5%
Non-converted (n=3)	5.6%
Converted (n=1)	1.9%

*Two acutely converted patients were removed from this calculation

**Dead patients within the 15-year follow-up period. Their deaths were not associated with a bariatric-metabolic surgical procedure

Abbreviations: *GERD* gastroesophageal reflux disease, *SG* sleeve gastrectomy, *RYGB* Roux-en-Y gastric bypass, *R* range

Four patients died within the 15-year follow-up period; however, their deaths were not associated with any bariatric-metabolic surgical procedure. These four patients and one patient who was acutely converted for early leak were excluded from further analysis in terms of weight loss and improvement of AMP. Another acutely converted patient, who is one of the four deceased patients, died more than 12 years after the conversion.

A complete follow-up after 15 years was attained in 46 of the remaining 48 patients (95.8%). The median follow-up period was 186 months (range 181–207 months) ≙15.5 years.

### Conversion Rate and Weight Loss

**Fig. 2:**
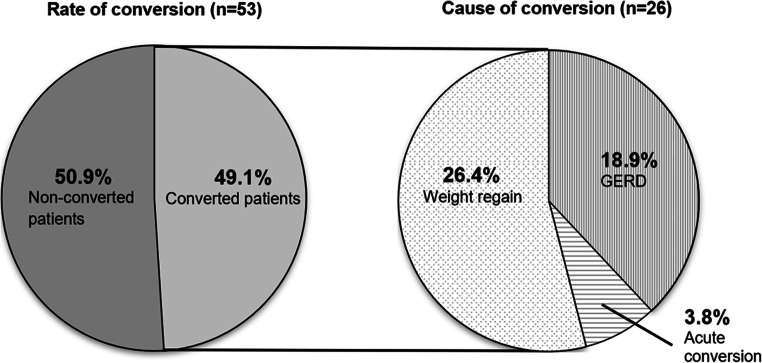
Conversion rate and cause of conversion 15 years after sleeve gastrectomy. Abbreviations: GERD gastroesophageal reflux disease

A total of 26 (49.1%) of 53 patients were converted: 25 to Roux-en-Y gastric bypass (RYGB) and one to biliopancreatic diversion with duodenal switch. The main indication for the conversion was WR in 14 patients (26.4%), symptomatic GERD in ten patients (18.9%), and acute conversion for an early leak in two patients (3.8%); see Table 1 and Fig. 2. Twelve of the converted patients (46.2%) in fact suffered from both, WR and GERD.

Mean weight and BMI at the time of the SG were 136.8 ±27.8kg and 48.7 ±9.2kg/m^2^. The history of weight within the 15-year follow-up period, excluding deceased and acutely converted patients, is presented in Table 2. The lowest postoperative weight and BMI these patients were able to reach after a median of 12 months were 86.3 ±19.8kg/m^2^ and 30.9 ±6.2kg/m^2^, representing a total weight loss (TWL) of 36.9 ±11.7.

**Table 2:** Patients’ history of weight

	All patients* (n=48)	Non-conversion* (n=24)	Conversion* (n=24)	
			Weight regain *(n= 14)*	GERD *(n= 10)*
SG (n=48)				
Mean age OP (years)	38.8 ±12.5	39.5 ±13.5	35.7 ±12.9	40.1 ±8.1
Weight (kg)	136.8 ±27.8	135.7 ±28.6	140.4 ±31.3	133.4 ±21.4
BMI (kg/m^2^)	48.7 ±9.2	49.5 ±9.9	47.9 ±9.6	46.7 ±6.2
Nadir (n=48)				
Weight (kg)	86.3 ±19.8	85.5 ±20.2	89.7 ±19.3	84.6 ±20.8
BMI (kg/m^2^)	30.9 ±6.2	31.4 ±6.7	30.5 ±5.3	29.6 ±6.0
Change in BMI (kg/m^2^)	17.8 ±8.4	18.1 ±8.6	17.4 ±8.8	17.1 ±8.3
EWL (%)	71.6 ±23.3	70.8 ±22.8	72.3 ±22.3	73.0 ±28.0
TWL (%)	36.9 ±11.7	37.0 ±9.5	36.1 ±13.9	36.6 ±14.2
Conversion (n=24)				
Weight (kg)			119.8 ±23.7	102.6 ±23.3
BMI (kg/m^2^)			40.7 ±5.4	35.8 ±6.3
Change in BMI (kg/m^2^)			7.2 ±8.7	10.9 ±8.4
EWL (%)			23.7 ±31.5	44.8 ±32.2
TWL (%)			14.7 ±16.5	23.1 ±18.3
>10 years (n=49)** [13]	(n=49)	(n=32)	(n=11)	(n=6)
Weight (kg)	98.1 ±21.3	100.8 ±22.1	98.0 ±20.5	80.0 ±5.9
BMI (kg/m^2^)	35.5 ±7.0	36.4 ±7.4	34.7 ±5.7	28.7 ±4.7
Change in BMI (kg/m^2^)	13.8 ±10.0	13.9 ±10.1	9.9 ±6.4	15.3 ±5.0
EWL (%)	54.0 ±26.7	52.5 ±24.8	52.8 ±32.7	73.5 ±20.2
TWL (%)	28.7 ±14.3	26.5 ±13.1	26.5 ±15.8	42.4 ±16.2
>15 years (n=46)				
Weight today (kg)	92.4 ±20.8	93.0 ±22.1	91.8 ±15.9	91.4 ±24.5
BMI today (kg/m^2^)	33.1 ±6.4	34.3 ±6.9	31.5 ±5.2	31.5 ±6.2
Change in BMI (kg/m^2^)	15.6 ±9.9	15.2 ±9.8	17.1 ±10.3	15.2 ±10.6
EWL today (%)	61.0 ±24.8	57.7 ±22.9	67.3 ±29.4	63.1 ±25.7
TWL today (%)	32.5 ±13.7	31.5 ±12.6	34.6 ±14.8	31.5 ±16.2

*Deceased and acutely converted patients (n=5) were removed from this calculation

**Results 10 years after SG are based on the previous publication [13] of the same patient collective

Abbreviations: *GERD* gastroesophageal reflux disease, *SG* sleeve gastrectomy, *BMI* body mass index, *EWL* excess weight loss, *TWL* total weight loss

**Fig. 3:**
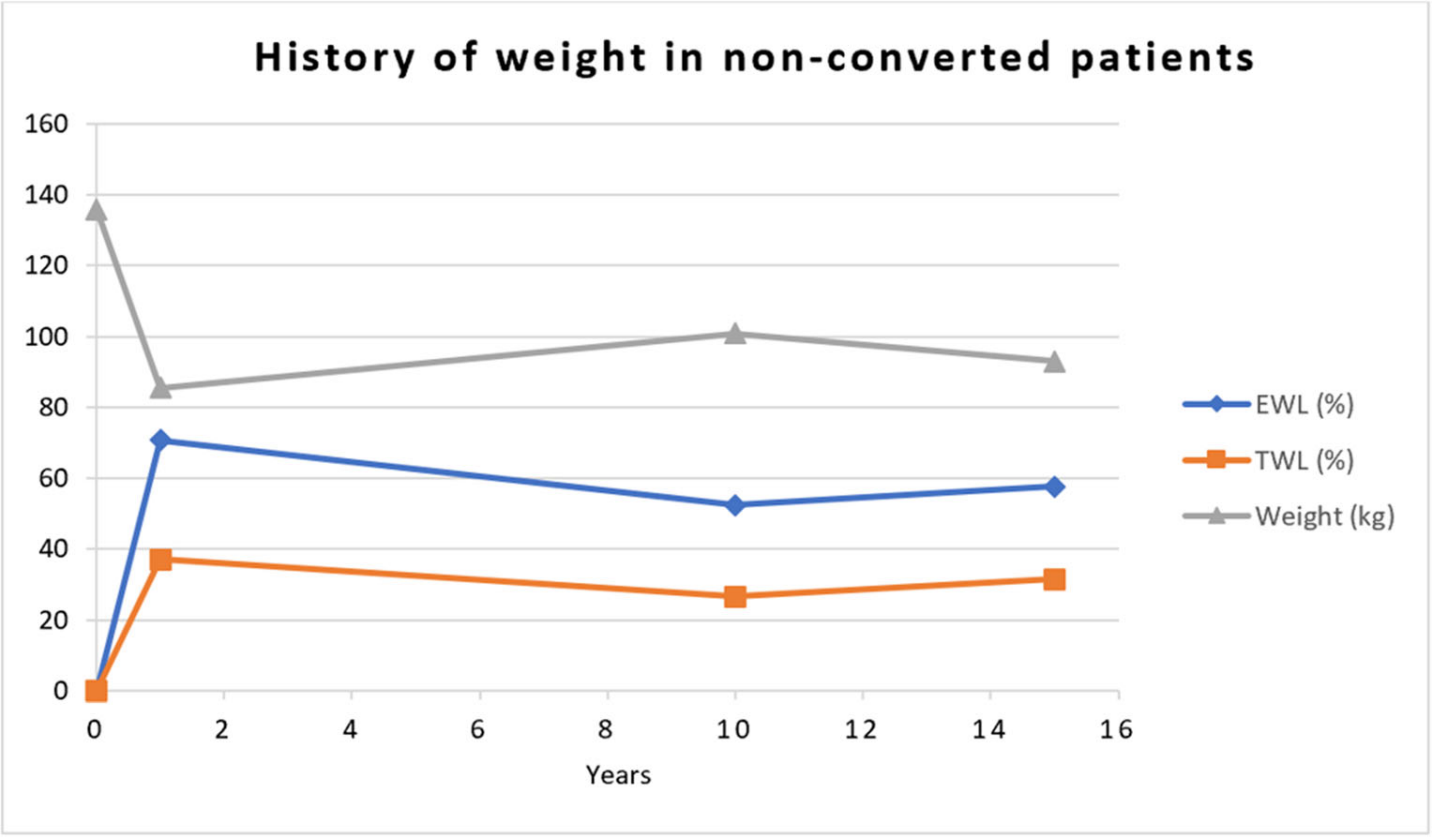
Weight, EWL, and TWL in non-converted patients over the time period of 15 years

Weight and BMI at the time of the conversion in the entire group of converted patients were 112.0 ±24.6kg and 38.4 ±6.2kg/m^2^ after a median period of 48 months. After a follow-up of 186 months, the non-converted group was able to reach a weight, BMI, and TWL of 93.0 ±22.1kg/m^2^, 34.3 ±6.9kg/m^2^, and 31.5 ±12.6%. The weight, BMI, and TWL of the converted group was 91.6 ±19.3kg/m^2^, 31.5 ±5.5kg/m^2^, and 32.9 ±15.1%. A detailed history of weight in patients converted mainly due to reflux or weight regain is highlighted in Table 2. To highlight weight changes over time, data from our previous publication based on a 10-year follow-up [13] was added to Table 2. The history of weight in non-converted patients over the period of 15 years is highlighted in Fig. 3.

### Remission of Associated Medical Problems

The history of AMP, including improvement and new onset, is shown in Table 3. At the time of the SG, 12 (25.0%) of 48 patients had arterial hypertension (AH), one (2.1%) had diabetes mellitus type II (DMII), two (4.2%) had hyperlipidemia (HL), one (2.1%) had obstructive sleep apnea (OSA), and three (6.3%) had diseases of bones and joints (DBJ). Within the 15 years of follow-up, the remission rate for AH was 37.5% in the non-converted group and 75.0% in the converted group. Further remission rates of DMII, HL, OSA, and DBJ are presented in Table 3.

**Table 3:** Patients’ associated medical problems 15 years after SG

	All patients* (n=48)	Non-conversion* (n=24)	Conversion* (n=24)
No associated medical problems (SG)	34/48 (70.8%)	19/24 (79.2%)	15/24 (62.5%)
No associated medical problems (15 years)	36/48 (75.0%)	19/24 (79.2%)	17/24 (70.8%)
Arterial hypertension (SG)	12/48 (25.0%)	8/24 (33.3%)	4/24 (16.7%)
Remission (15 years)	6/12 (50.0%)	3/8 (37.5%)	3/4 (75.0%)
New onset (15 years)	2/36 (5.6%)	1/16 (6.3%)	1/20 (5.0%)
Diabetes mellitus II (SG)	1/48 (2.1%)	1/24 (4.2%)	0
Remission (15 years)	0	0	0
New onset (15 years)	1/47 (2.1%)	1/23 (4.3%)	0
Hyperlipidemia (SG)	2/48 (4.2%)	1/24 (4.2%)	1/24 (4.2%)
Remission (15 years)	0	0	0
New onset (15 years)	1/46 (2.2%)	0	1/23 (4.3%)
Obstructive sleep apnea (SG)	1/48 (2.1%)	0	1/24 (4.2%)
Remission (15 years)	1/1 (100%)	0	1/1 (100%)
New onset (15 years)	0	0	0
Diseases of bones and joints (SG)	3/48 (6.3%)	1/24 (4.2%)	2/24 (8.3%)
Remission (15 years)	0	0	0
New onset (15 years)	2/45 (4.4%)	0	2/22 (9.0%)

*Deceased and acutely converted patients (n=5) were removed from this calculation

Abbreviations: *SG* sleeve gastrectomy

New diagnoses of AH, DMII, HL, OSA, and DBJ within the 15 years of follow-up were observed in 5.6%, 2.1%, 2.2%, 0%, and 4.4%, respectively.

### Outcome and Patients’ Quality of Life

**Fig. 4:**
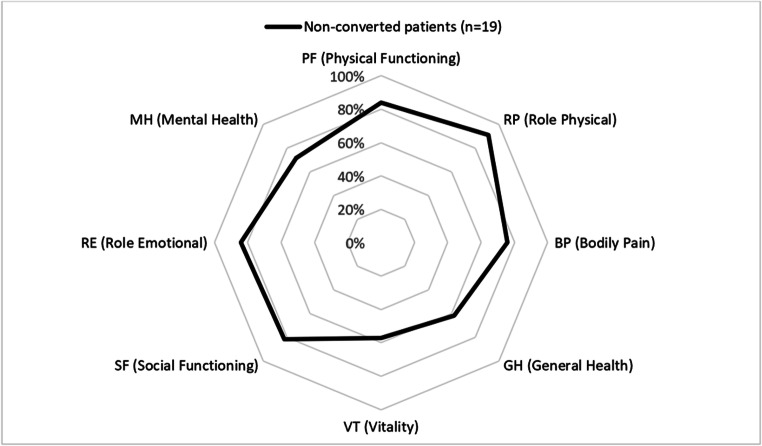
Short Form 36 (SF-36) in non-converted patients 15 years after sleeve gastrectomy

Outcome scores and QOL of non-converted patients after 15 years are highlighted in Table 4. The outcome scores were completed by 19 of 24 (79.2%) non-converted patients (excluding patients that died within the follow-up period). The mean BAROS after 15 years was 2.9 ±2.1, which equals a fairly efficient outcome, and the patients’ BQL score was 52.9 ±9.7. Results in the different categories of the SF-36 score are highlighted in Table 4 and Fig. 4. Data from our two previous publications based on a 10-year follow-up [12, 19] was added to Table 4 to highlight changes in outcome and QOL scores.

**Table 4:** Quality of life in non-converted patients

	10 years*	15 years
	(n=48)	(n=19)
BAROS	2.0 ±1.9	2.9 ±2.1
BQL	48.2 ±9.8	52.9 ±9.7
SF-36		
PF (physical functioning)	78.2 ±22.4	83.9 ±11.6
RP (role physical)	75.6 ±37.2	90.8 ±20.8
BP (bodily pain)	72.0 ±30.4	75.5 ±28.7
GH (general health)	60.1 ±20.9	62.2 ±21.1
VT (vitality)	55.5 ±22.0	57.4 ±24.3
SF (social functioning)	79.9 ±27.3	82.3 ±25.7
RE (role emotional)	72.1 ±41.7	84.2 ±34.0
MH (mental health)	68.7 ±21.9	71.6 ±22.1

*Results 10 years after SG are based on two previous publications [12, 19] of a larger sample size of the same patient collective

Abbreviations: *BAROS* Bariatric Analysis and Reporting Outcome System, *SF-36* Short Form 36, *BQL* Bariatric Quality of Life

## Discussion

This multicenter study presents a follow-up of at least 15 years after SG in terms of weight loss, conversion rate, remission of AMP, QOL, and data from bariatric-metabolic outcome scores. To the authors’ best knowledge, this study represents the longest follow-up after SG in the literature so far with a high follow-up rate. As mentioned earlier, this study is an update of the exact same patient collective published 5 years ago, which presented a follow-up of 10 years after SG [13]. Therefore, the current study is well-suited to compare the outcome at both points in time and to track a trend in the long-term follow-up.

### Conversion Rate and Weight loss

Data on a long-term outcome of more than 10 years focusing on weight loss after SG are rare in the literature. However, comparing this study’s data (15 years follow-up) to the previous study with a 10-year follow-up, an interesting trend may be observed. Long-term weight loss remains stable or even decreases, however, does so at the cost of a higher conversion rate. To be exact, mean weight of the total collective decreases from 98.1 (after 10 years) to 92.4kg (after 15 years), whereas the conversion rate increases from 35.8 (after 10 years) to 49.1%. This shift of patients with weight regain from the non-converted group to the converted group with further weight loss leads to better results in both groups. Also, the mean EWL of the total collective increases from 54.0 (after 10 years) to 61.0% (after 15 years). The high number of additional conversions within the last 5 years was, in a manner of speaking, in part caused by our previous study [13]. As patients were invited to our bariatric center, those suffering from weight regain and/or reflux, who might not have been inclined to do so otherwise, asked for solutions to cure their condition. Some of them ended up being converted; however, none of them were in any way urged to make this decision. Additionally, it must be considered that patients in the converted group had at least two bariatric-metabolic operations.

There is a small number of studies on SG with a follow-up of at least 10 years available in the literature today. Chang D.M. et al. presented 65 patients after SG that reached a long-term follow-up of 10 years. The authors report an EWL of 70.5% with a low conversion rate of only 16.9%; however, the follow-up rate was only 64.4% [9]. Another study of 110 patients after SG with 11.7 years follow-up by Arman G.A. et al. reported an EWL of 62.5%, which is equal to the data found in the present study. The conversion rate was 25.0%, but a low patient follow-up rate of only 59.1% was reached. Castagneto Gissey L. et al. presented their 114 patients 10–11 years after SG with quite a high follow-up rate of 77%. Their outcomes were an EWL of 52.5%, which is comparable to our previously published study [13] and a very low conversion rate indeed of only 2% [8].

It may be summarized that long-term studies after SG showed a relatively stable weight and acceptable weight loss rate, and an increasing conversion rate over time.

### Remission of Associated Medical Problems

The current study presents the first remission rates and rates of new onsets of AMP at more than 15 years after SG. Nevertheless, the description is based on a relatively small population as only 29.5% of the patients had at least one associated medical problem at the time of the SG. Therefore, the validity of the data based on AMP is limited.

Regardless of the small population, the improvement rates are relatively stable over time with only small numbers of new onsets compared to our 10-year outcome study [13]. This has to be seen as an achievement as this patient population is at least 15 years older than their mean age at the time of the SG, especially considering the fact that the higher the age of the patient, the more likely they are to develop new AMP.

Also, the remission rates of AMP after SG in other long term-studies with a follow-up of more than 10 years are based on small patient populations as well. In terms of DMII remission, rates between 39.1 by Chang D.M. et al. [9] and 64.7% by Castagneto Gissey L. et al. [8] after 10 years are reported. The remission rates of AH differ the most in the long-term follow-up in the literature: from 28.6 reported by Arman G.A. et al. [11] after 11.7 years to up to 78.4% after 10 years by Chang D.M. et al. [9]. The reported HL remission rates are 36.4% [8], 40.0% [11], and 51.3% [9], and remission rates of OSA are 66.0% [11] and 72.2% [8]. It should be noted, however, that the reported values are competitive rates of both converted and non-converted patients and therefore data does not exclusively reflect the long-term outcome after non-converted SG.

It can be summarized that acceptable to good remission rates of AMP after SG still exist after a long follow-up period of 10–15 years. Nevertheless, these outcomes are based on rather weak data due to small study cohorts. For more reliable data, the publication of randomized control trials (SM-BOSS and SLEEVEPASS) on long-term outcomes after SG has to be awaited—they currently report mid-term outcomes [20].

### Outcome and Quality of Life

The following QOL and outcome scores were used in the current study: BAROS as an outcome score after bariatric-metabolic procedures, BQL as a QOL questionnaire specifically created for patients with obesity after a bariatric-metabolic procedure, and SF-36 as a general QOL questionnaire reflecting a bigger picture of patients’ psychological and physical QOL.

A comparison of the BAROS score in today’s study to the data gained from the score in our previously published study 10 years after SG shows that this score has increased from 2.4 (10 years) to 2.9 (15 years) in non-converted patients after SG; nevertheless, the classification is still the same reflecting a fairly efficient outcome. The reason for the increased score is simply based on the high conversion rate, as patients converted due to weight regain were not included in this analysis and weight loss has a major impact on this score. A recently published study by Fiorani C. et al. reported a BAROS of 1.47 at an 8-year follow-up after SG [21].

Comparing data gained from the BQL and SF-36 scores in this study and our 10-year follow-up shows a slight improvement from 10 to 15 years after SG in the BQL as well as all eight categories of the SF-36 [19]. Again, this outcome reflects that weight regain and reflux have a major impact on patients’ QOL and, therefore, a conversion to RYGB may improve their QOL, if at least one of these side effects exists.

A recent study by Major P. et al. reports SF-36 data in 28 SG patients before the operation, at 1 year, and at 10 years after [22]. All 8 categories improved significantly in the first year but re-decreased 10 years after SG. Nevertheless, at 10 years after SG, the SF-36 QOL was still majorly improved in all categories compared to the preoperative data. The results 10 years after SG were very similar to those of our own study 10 years after SG [19].

To conclude, the best way to interpret patients’ QOL is comparing one patient collective’s data from different points in time, thus taking a longitudinal perspective. All the reported scores proved valid to work out the individual patient’s QOL at different points in time after SG.

### Limitations of the Study

This study presents a small collective from the beginning of SG as stand-alone procedure. The common operation technique has slightly changed since then, even though a worldwide standard does not exist, yet. To increase the number of patients, patients from other bariatric centers with the same operation technique at the time were included; however, the number of patients may still be too small to draw any final conclusions. Additionally, this also increases the number of surgeons who performed the procedure in these patients.

Another limitation is that 10 patients had gastric banding before SG, which may have affected weight loss and outcome. However, it should be considered that patients with previous bariatric-metabolic procedures reflect the typical collective of patients bariatric surgeons deal with. Also, the size of the bougie used in SG has changed slightly, which may lead to different weight loss outcomes in recently operated SG patients.

Finally, the current study lacks preoperative data on QOL; therefore, the collected data cannot be compared to patients’ QOL before SG.

## Conclusion

Fifteen years after SG, a good and stable postoperative weight was observed but at the cost of a high conversion rate of up to 49.1% due to weight regain and reflux. Patients converted to RYGB were able to achieve further weight loss and preserve good remission rates of AMP. SG in patients without the need of a conversion to another bariatric-metabolic procedure may be considered effective. In conclusion, a careful preoperative patient selection is mandatory when performing SG in patients with obesity.
